# Sex Differences in Functional Connectivity Patterns During Mental Rotation Task: An Event-Related EEG Study

**DOI:** 10.3390/bs16010144

**Published:** 2026-01-20

**Authors:** Shanshan Wang

**Affiliations:** School of Psychology, Hainan Normal University, No. 99 LongKun Nan Avenue, Haikou 571158, China; shanshanwangdelia@163.com

**Keywords:** sex differences, mental rotation, ERP study, functional connectivity

## Abstract

Numerous studies have shown sex differences in mental rotation (MR) tasks, but few have explored the internal neural influences through functional connectivity outcomes. To investigate neuro-activity influences on sex differences, this study conducted a revised MR task, examining low-scoring individuals via behavioral and electrophysiological measures. It obtained event-related potential (ERP) components from fronto-central channels and explored the functional connectivity of different frequency bands. The results showed males outperformed females, consistent with prior research. There were significant differences between the two sexes when completing the task successfully. Males had shorter response times, smaller ERP amplitudes, and stronger beta and gamma functional connectivity than females. Compared to females, males showed better behavioral performance, weaker fronto-parietal ERP activity, required less mental effort, and had more effective internal regulation in connectivity. This helps clarify the fundamental neural activities in MR between different sex groups.

## 1. Introduction

Mental rotation (MR) involves the cognitive transformation of mental imagery through rotation (Gardony et al., 2017), requiring participants to mentally identify, compare, and align figures presented in different orientations to match a reference figure (Sharma et al., 2019). Previous studies reveal that sex differences, variations in brain activity, hemispheric lateralization, strategy selection, dimensionality effects (two-dimensional vs. three-dimensional), and stimulus rotation angle remain debated topics within MR research. It has been established that MR encompasses spatial perception, spatial intelligence, visual imagery formation, spatial attention, working memory, decision-making, and abstract reasoning (Prescott et al., 2010; Sharma et al., 2019). Elucidating the underlying mechanisms of MR holds significant implications for diverse fields, including education, cognitive psychology, and clinical neuroscience (Jouzizadeh, 2024).

Research has established well-documented sex differences in mental rotation (MR) abilities, with males demonstrating significantly higher behavioral scores (Collins & Kimura, 1997; Quaiser-Pohl et al., 2006), faster figure rotation times (Bryden et al., 1990; Kail et al., 1979), and greater accuracy (Geary et al., 1992) than females. Beyond behavioral findings, numerous studies have reported sex differences in MR processing from various neurocognitive perspectives. For instance, sex-specific activation patterns in cortical regions during MR tasks are well-established (Gur et al., 2000; Nopoulos et al., 2000; Speck et al., 2000; Siegel-Hinson & McKeever, 2002), suggesting that males employ holistic strategies while females utilize analytic strategies (Jordan et al., 2002; Butler et al., 2006; Hugdahl et al., 2006). Studies have found significantly stronger activation in frontal areas among females (Butler et al., 2006; Hugdahl et al., 2006; Rubia et al., 2010) and in parietal areas among males (Butler et al., 2006; Hugdahl et al., 2006) during these tasks. Specifically, females exhibited stronger bilateral activation than males in the inferior temporal gyrus, the caudal part of the dorsal premotor area, the right superior parietal lobule, the intraparietal sulcus, the left inferior parietal lobule, and the anterior intraparietal sulcus. Conversely, males demonstrated stronger bilateral activation in the primary motor cortex, the left posterior intraparietal sulcus, and the right parieto-occipital sulcus (Jordan et al., 2002).

By reviewing the findings of sex differences in magnetic resonance (MR) studies, the frontal and parietal regions are engaged in visuospatial processing, with frontoparietal connectivity serving a critical role in supporting this function (Jouzizadeh, 2024). The frontoparietal network (FPN) serves as the central functional hub for cognitive control within the brain. Its core functions encompass the following:

(1) Acting as a flexible integration hub for cognitive control, facilitating whole-brain resource allocation during task adaptation and execution through robust functional connections with multiple networks, including the default mode network and the dorsal attention network.
(2) Constituting the neural substrate of fluid intelligence, wherein the degree of its coupling and functional integration strength with other brain networks significantly predicts individual intelligence scores.
(3) Regulating high-level executive functions, including working memory, reasoning, decision-making, cognitive flexibility, and problem-solving. Specifically, the left FPN primarily mediates language processing and memory, whereas the right FPN governs cognitive inhibition and visuospatial attention.
(4) Mediating decision-making and selection processes by encoding value signals through frontoparietal connections, weighing costs against benefits, and supporting goal-directed behavior (Smith et al., 2009; Yin et al., 2022).

Consequently, the current study has posited that the FPN is crucial for MR. To elucidate sex differences in MR task performance, it is proposed that integrating interpretations of brain activity patterns and electroencephalographic (EEG) band activities may offer enhanced explanatory power for comprehending the intrinsic mechanisms underlying sex differences.

Regarding differences in oscillatory activity during mental rotation (MR) tasks, notable findings have also been observed within frontal and parietal regions. Concerning the functional significance of band-specific activity, prior research has established that theta oscillations are associated with stimulus perception in visual tasks, attentional resource allocation, and memory processes (Busch et al., 2009; Dravida et al., 2019). During visual imagery elicited by external stimuli, theta and beta band activity in central, frontocentral (Hiew et al., 2023; Trambaiolli et al., 2019), and frontoparietal regions (Seguin et al., 2023) have been linked to the perception of object motion (Borst et al., 2012), motor imagery, and navigation processing. The alpha band exhibits sensitivity to cognitive task demands, such as those involving visual imagery (Salenius et al., 1995) and mental visuospatial operations (Neubauer et al., 2006; Neubauer & Fink, 2009). Frontal alpha oscillations are particularly sensitive to higher-order cognitive task performance and mental effort (Akiyama et al., 2017; Neubauer et al., 2010; So et al., 2017; Ter Horst et al., 2013). Gamma-band activity has been demonstrated to play a critical role in the mental rotation of three-dimensional objects (Engel & Singer, 2001; Wyart & Tallon-Baudry, 2008) and in visual cognition (Hoogenboom et al., 2006; Iwaki et al., 2011), and is postulated to reflect the integration of information distributed across distinct cortical regions (Bressler, 1990).

Numerous studies have established associations between theta, alpha, beta, and gamma band activities and mental rotation (MR). Furthermore, distinct patterns of band activity have revealed sex differences across various processing stages during MR tasks. For instance, theta band connectivity exhibits enhancement in the frontal region for females and in the lateral regions for males; conversely, alpha band connectivity demonstrates reduction in the frontal region for females and enhancement in the frontal region for males (Gootjes et al., 2008). Within the left prefrontal and parietal regions, females display increased theta band activity, whereas males exhibit increased gamma band activity (Jouzizadeh, 2024). Females demonstrate greater frontal theta synchronization, while males show greater frontal theta desynchronization (Jaušovec & Jaušovec, 2012). Regarding network connectivity, females exhibit greater theta coherence in the right prefrontal, central, and frontocentral regions, while males display higher gamma coherence in the left prefrontal and left parietal regions (Jouzizadeh, 2024). For intra-hemispheric coherence, females manifest stronger connectivity (indicating greater consistency) in the beta and theta bands within the right hemisphere. For inter-hemispheric coherence, males demonstrate increased connectivity in the alpha and gamma bands across regions (including the frontal and parietal lobes (Jouzizadeh, 2024).

Integrating the MR task differences indicates that females recruit frontal resources more extensively, whereas males engage parietal resources to a greater degree. This finding supports the proposition that distinct genders employ different cognitive strategies. The current investigation will integrate the fronto-parietal network and diverse frequency bands to explore sex differences in MR. Given the significant contribution of EEG variability, event-related potentials (ERP) will be utilized. Based on previous research conclusions, we hypothesize that within the MR task, males will demonstrate superior behavioral performance compared to females. However, regarding functional connectivity (FC), females demonstrate enhanced FC within the frontal region, while males exhibit enhanced FC within the parietal region. Integrating prior findings and the psychological phenomena associated with theta, alpha, beta, and gamma frequency bands, this research will specifically focus on the characteristics of these bands within FC during the analysis. The results of this study elucidate specific sex differences and the differential characteristics of FC during the visuospatial transformation task via brain source localization combined with the mental rotation paradigm.

## 2. Method

### 2.1. Participants

Sixty-eight participants (35 female) were recruited from the University (M = 19.16 years, SD = 1.19; range = 18–23 years). All participants were native Mandarin speakers with normal or corrected-to-normal vision. The Ethics Committee of the University approved this research, which was conducted in compliance with relevant guidelines. Before the experiment, all participants provided written informed consent to participate voluntarily.

### 2.2. Materials and Procedure

#### 2.2.1. Materials

In mental rotation (MR) research, previous investigations into the influence of object dimensionality (2D vs. 3D) on rotation speed indicate minimal impact (Shepard & Metzler, 1988). It has been posited that 3D objects represent 2D planar figures augmented with thickness, thereby increasing their structural complexity (Jolicoeur et al., 1985). However, if these added thicknesses are not perceived as integral to the object’s three-dimensional structure, they may not substantially elevate its perceived complexity. Notably, within MR tasks, employing familiar objects as standard stimuli, the effect of object dimensionality is further attenuated (Just & Carpenter, 1976; Shepard & Metzler, 1988; Tarr & Pinker, 1989).

Furthermore, research in MR has demonstrated that the rotation angle significantly influences performance. Evidence suggests that differential rotation rates and strategic approaches are adopted for acute vs. obtuse angles. Gender differences have also been observed. Specifically, studies indicate that for angular disparities below 90°, mental rotation constitutes the default strategy and is executed with relative ease, as reflected in higher accuracy rates for such angles (Boone & Hegarty, 2017). Consequently, the cognitive effort required for rotation is typically less than the combined effort needed to switch to and execute an alternative strategy. Conversely, for angles exceeding 90°, the cognitive load associated with mental rotation may increase substantially, potentially making the combined effort of switching to and applying an alternative strategy comparatively lower. This suggests participants dynamically adjust their strategies based on the relative efficiency and task difficulty associated with different approaches. Rotation remains the most effective and successful strategy for smaller angular disparities, whereas alternative strategies may prove more efficient for larger angles, particularly if better suited to the overall task demands (Boone & Hegarty, 2017). Additional studies report that variations in dimensional representations and rotation angles lead to distinct rotation rates, facilitating adaptation to angular disparities, particularly those between 60° and 180° (Jolicoeur et al., 1985).

Based on the summaries, it was observed that in the MR task, an interactive effect exists between dimensions and rotation angles. Simultaneously, this influence prompts participants to adopt varied strategies to complete the task in response to the rotation angle challenge, with sex differences also evident. However, these differences are not the primary focus of this study for further investigation. Consequently, this study balanced the issues pertaining to dimensions and angles and implemented specific adaptations to the MR task.

First, given that the impact of the dimension issue is pronounced only when individuals lack familiarity with certain objects, the study presented stimuli consisting of images of highly familiar objects. This ensured that individuals’ perception of the complexity of their 3D forms was minimal, equivalent to that of 2D presentations. Therefore, to balance the dimension issue, we employed 2D highly familiar objects as stimuli.

Second, to balance the interactive effect of rotation angles and dimensions and to mitigate sex differences, the study partitioned the stimuli into distinct components. Subsequently, the forms of each component were presented simultaneously at 45°, 135°, 225°, and 315° rotation angles.

#### 2.2.2. Procedure

**Figure 1:**
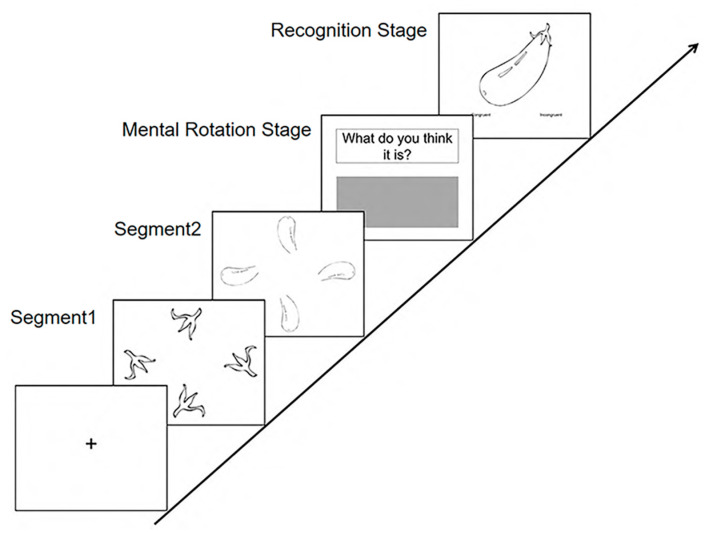
Procedure of the experiments.

Based on the study’s objectives, the experimental task procedure was structured as follows. Original stimuli comprised line drawings depicting common objects (e.g., vegetables, vehicles, and animals), rendered with black outlines. These stimuli were segmented into two or three non-meaningful fragments. Each fragment was presented in four distinct rotational orientations within a single slide (Figure 1; see Segments 1 and 2).

Each trial commenced with a black fixation cross displayed centrally on a white background for 800 ms. Subsequently, each slide containing a fragmented segment was presented for 1000 ms. Following the presentation of all segments, a prompt (“What do you think it is?”) appeared at the top of the screen. Participants were required to input their response within a designated grey dialog box below the prompt within a 10 s response window (Figure 1; see Mental Rotation Stage (including rotation and composition)). Responses necessitated precise identification of the object, excluding general or ambiguous categorizations (e.g., “an animal” or “a vegetable”). Upon entering their response, participants pressed the ‘ENTER’ key. This action triggered the immediate display (within 2 s) of the original, intact figure alongside two recognition options. Participants indicated their recognition judgment by pressing ‘1’ for “it is the same as my answer or I guessed” or ‘2’ for “it is different from my answer” (Figure 1; see Recognition Stage). Each procedural step is labeled and visually represented in Figure 1.

A total of 160 trials were carried out, randomly mixed across four blocks of 40 trials per block. Eighty figures were split into two segments, and the remaining 80 figures were split into three parts. Each block had the same number of figures with split segments. Five practice trials were completed before the formal experiment. All stimuli appeared on the 19 in CRT monitor with 85 Hz refresh rates, 0.1° spatial resolution and 1024- by 768-pixel resolution, viewing at a fixed distance of approximately 60 cm.

## 3. EEG Recordings and Data Reduction

Electroencephalography (EEG) data were recorded at 64 scalp sites, using tin electrodes mounted in an elastic cap (Brain Products, Munich, Germany), at a 500 Hz sampling rate. The references were the left and right mastoids and a ground electrode at the medial frontal aspect. The vertical electrooculograms (EOGs) were recorded supra- and infra-orbitally with respect to the right eye. The horizontal EOG was recorded from the left vs. the right orbital rim. EEGs and EOGs were amplified using a 0.05–100 Hz bandpass. All electrode impedances were kept below 5 kΩ.

Offline, the data were re-referenced to the average of the left and right mastoids, and a bandpass filter of 0.1–40 Hz was applied. Trials with horizontal EOG voltage exceeding ±30 μV, and those contaminated with artifacts due to amplifier clipping and peak-to-peak deflection exceeding ±80 μV, were excluded from the average. The EEG data for the Mental Rotation Stage were segmented into 2000 ms duration epochs, while the EEG data for viewing stimuli were segmented into 800 ms epochs, all including a 200 ms pre-stimulus baseline recording. Additionally, an independent component analysis (ICA) was used to remove ocular artifacts (blinks and saccades), and artifact rejection was performed to exclude effects of muscle or recording artifacts and excessive noise.

## 4. Data Analysis

### 4.1. Behavioral Analysis

It was a 2 × 2 (Solution with different kinds of answers: Unsuccessful MR with incorrect answers vs. Successful MR with correct answers) × (Group: Female vs. Male) mixed factorial design, and the interaction effect were analyzed, in which Group was a between-subject variable, and Solution was a within-subject variable. The data of reaction times (RTs) were analyzed with independent samples *t*-tests. The *p*-value for significance (*p* < 0.05) was reported.

### 4.2. ERP Analysis

To test the processing traits, the EEG data from the Mental Rotation Stage were analyzed. P300 (150–300 ms) in the fronto-central regions (F7, F5, F3, F1, FZ, F2, F4, FC3, FC1, FCZ, FC2, FC4, C3, C1, CZ, C2, and C4) of the groups were measured. Specifically, Group (Female vs. Male) × Solution (Unsuccessful vs. Successful) repeated measures analyses of variance (ANOVA) were assessed to determine mean amplitude effects for each component of interest.

The *p*-values of all main and interaction effects were adjusted using sphericity violations and the Greenhouse–Geisser correction for repeated-measure effects. When significant interactions emerged, data were analyzed with a Bonferroni correction, and Student *t*-tests or one-way ANOVAs were utilized for within simple effects analyses. All analyses were performed using SPSS (v 22.0). The current study mainly focused on the differences between groups, so that, while there were significant interactions, they were not relevant to the current research focus and thus not reported.

### 4.3. FC Analysis

Connectivity analysis. For neural source estimation, the Brainstorm software (Version 3.240829; Tadel et al., 2011) was employed to generate a stable and conservative inverse solution. The head model was constructed using the OpenMEEG Boundary Element Method (BEM) (Gramfort et al., 2010), incorporating the scalp, outer skull, and inner skull. Cortical reconstruction for the average MRI was performed based on the ICBM152 MNI templates. A noise covariance model was implemented to enhance neural activity localization accuracy, utilizing fixed source orientation and depth weighting. The depth weighting parameters were set with an exponent of 0.5 and a weight limit of 10.

The functional connectivity (FC) analysis was conducted to mitigate the volume conduction problem inherent in EEG recordings. Averaged EEG signals, within the 150–300 ms post-stimulus onset window, were selected as representative of functional connectivity. The extracted inversion kernel derived via standardized low-resolution brain electromagnetic tomography (sLORETA) was multiplied to obtain signals at the nodes and edges of the FC network. These network elements are represented by the triangular mesh generated using the BEM (Klados et al., 2013).

The weighted phase-lag index (wPLI) was selected for constructing complex brain networks in this study. The wPLI quantifies the consistency of phase differences between oscillatory brain source signals (Vinck et al., 2011). The wPLI values were computed for all pairwise combinations of the 62 sources within the theta (5–7 Hz), alpha (8–12 Hz), beta (15–29 Hz), gamma1 (30–59 Hz), and gamma2 (60–90 Hz) frequency bands. This resulted in individual connectivity matrices (62 × 62) for each participant. The relative phase of the source signals was determined using the Hilbert transform.

Two-sample *t*-tests were conducted on wPLI across selected time windows and frequency bands to examine connectivity differences using the Network-Based Statistic (NBS) software (Version 1.2; Zalesky et al., 2010). Networks exhibiting the strongest connections were visualized using the BrainNet toolbox (Version 1.61; Xia et al., 2013). To assess overall connectivity changes, a univariate connectivity measure was derived by applying principal component analysis (PCA) to the connectivity matrices and correlating this measure with individual connectivity matrices.

The significance threshold was set at *p* < 0.01, using 5000 permutations with false discovery rate (FDR) correction applied. Statistical parametric maps were visualized using MRIcron (Version 4.0). Complex network analysis was performed on all electrode pairs, employing electrodes as nodes and the inverse wPLI values as edge weights.

## 5. Results

### 5.1. Behavioral Results

The repeated ANOVA of reaction time (RT) for Group × Solution was measured. It found the significant main effect for Group (F(1,66) = 5.11, *p* = 0.27, ŋ_p_^2^ = 0.07) and Solution (F(1,66) = 137.78, *p* < 0.001, ŋ_p_^2^ = 0.68). Further analysis demonstrated that the solution when mental rotation was unsuccessful spent significantly more time than when mental rotation was successful (Successful: M ± SD = 4010.74 ± 125.95; Unsuccessful: M ± SD = 4839.08 ± 147.65), and females spent significantly more time than males in both solutions (all *p*-values < 0.05; see Table 1).

### 5.2. ERP Results

**Figure 2:**
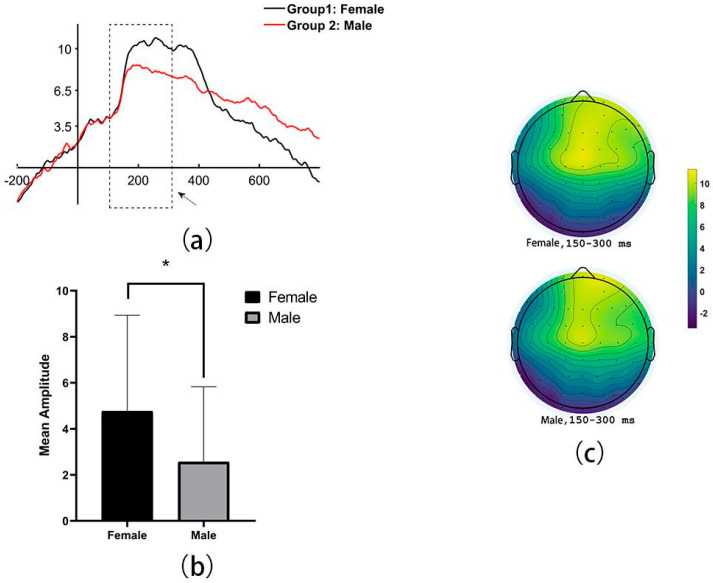
The sex differences of the grand average amplitude when MR is successful: (**a**) the line distribution (Note. The dashed box means the duration of 150–300 ms), (**b**) the bar chart of the specific difference between groups (Note. * <0.05), and (**c**) the scalp distribution.

Based on the design of the experiments and exploring the results, it was found that the significant group differences in mean amplitude of ERP were set within a duration of 150–300 ms during the processing of mental rotation successfully. Specifically, it revealed significantly larger mean amplitude of female than of male, during successful MR processing in the fronto-central areas (including electrodes: F7, F5, F3, F1, FZ, F2, F4, FC3, FC1, FCZ, FC2, FC4, C3, C1, CZ, C2, and C4, *t* = 2.42, *p* = 0.02, 95% IC = [0.38,4.02]; Female: M ± SD = 4.76 ± 4.17; Male: M ± SD = 2.56 ± 3.27, See Figure 2).

### 5.3. FC Results

All electrodes, as the sources of wPLI, were computed for the functional connectivity, acquiring the average connectivity by a 62 × 62 matrix. After conducting NBS analyses with two-sample *t*-tests for both frequency bands (theta, alpha, beta, gamma1, and gamma2: whole epochs, 150–300 ms), the significant functional connectivity in the beta, gamma1 and gamma2 emerged.

**Figure 3:**
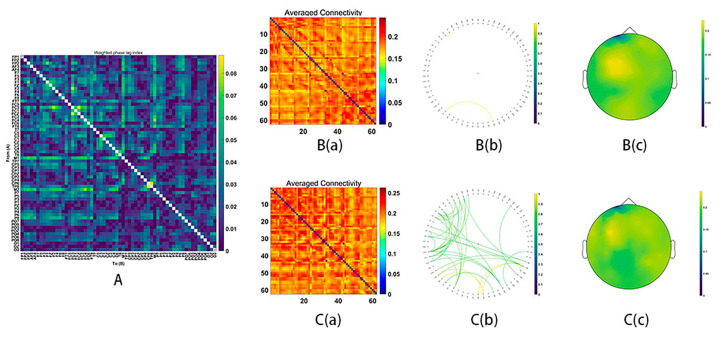
Functional connectivity in the beta frequency band (15–29 Hz). (**A**) The average connectivity of the significant differences between groups, measured by the weighted phase-lagging index (wPLI). (**B**) The female group. (**B**(**a**)) Matrix of average connectivity. (**B**(**b**)) The significant connectivity lines. (**B**(**c**)) The scalp distribution. (**C**) The male group. (**C**(**a**)) Matrix of average connectivity. (**C**(**b**)) The significant connectivity lines. (**C**(**c**)) The scalp distribution.

**Figure 4:**
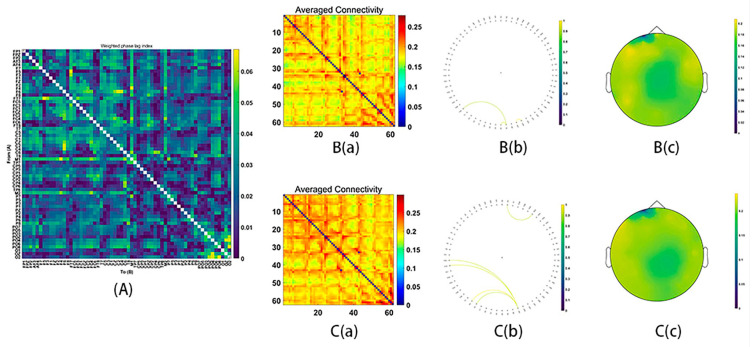
Functional connectivity in the gamma1 frequency band (30–59 Hz). (**A**) The average connectivity of the significant differences between groups, measured by the weighted phase-lagging index (wPLI). (**B**) The female group. (**B**(**a**)) Matrix of average connectivity. (**B**(**b**)) The significant connectivity lines. (**B**(**c**)) The scalp distribution. (**C**) The male group. (**C**(**a**)) Matrix of average connectivity. (**C**(**b**)) The significant connectivity lines. (**C**(**c**)) The scalp distribution.

**Figure 5:**
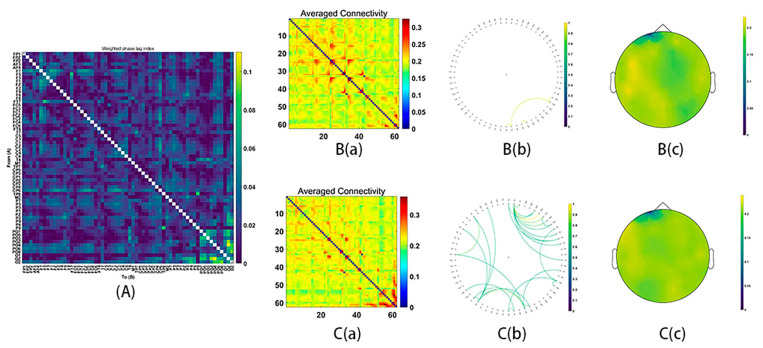
Functional connectivity in the gamma2 frequency band (60–90 Hz). (**A**) The average connectivity of the significant differences between groups, measured by the weighted phase-lagging index (wPLI). (**B**) The female group. (**B**(**a**)) Matrix of average connectivity. (**B**(**b**)) The significant connectivity lines. (**B**(**c**)) The scalp distribution. (**C**) The male group. (**C**(**a**)) Matrix of average connectivity. (**C**(**b**)) The significant connectivity lines. (**C**(**c**)) The scalp distribution.

During MR processing with correct answers, beta (15–29 Hz), gamma1 (30–59 Hz) and gamma2 (60–90 Hz) network analysis revealed significant group differences (Beta: *p* = 0.03; gamma1: *p* = 0.01; gamma2: *p* = 0.02). It was found that stronger functional connectivity was present in the male group than in the female group. The functional network centered around the frontal and parietal cortex (beta bands, Figure 3; gamma1 bands, Figure 4; gamma2 bands, Figure 5).

## 6. Discussion

The results demonstrated significant sex differences, specifically when participants successfully completed the mental rotation (MR) task, as opposed to unsuccessful attempts. Specifically, females exhibited significantly longer response times compared to males. The ERP data revealed a significantly larger mean amplitude in the fronto-central region among females, and the functional connectivity (FC) analysis indicated significantly weaker FC in the frontal-parietal network for females within the beta and gamma frequency bands.

Previous research has established that males typically demonstrate superior performance on MR tasks, characterized by shorter response times and higher accuracy than females. Consistent with these findings, the present study observed significantly longer response times in females compared to males, but only during successful task completion and without significant accuracy differences. This study, however, revealed more nuanced distinctions. While disparities exist at the overall level, more pronounced sex differences emerge specifically when participants successfully solve the MR task and obtain correct responses. Reviews indicate that numerous brain regions are implicated in mental rotation (Zacks, 2008), including the strongly associated prefrontal and parietal cortices (Eisenegger et al., 2007; Ecker et al., 2008; Podzebenko et al., 2005; Koshino et al., 2005; Seurinck et al., 2005). Aligned with prior findings, this study identified significant sex differences in ERP components localized to the fronto-central region. Regarding the neural substrates, evidence indicates that prefrontal activation relates to comparing the target object with the mentally rotated representation (Carpenter et al., 1999), while parietal activation is associated with processing sensory input and generating mental rotation movements (Podzebenko et al., 2005). Furthermore, secondary motor areas (i.e., the premotor cortex and supplementary motor area) are implicated in computing rotation angles, matching objects, and executing final decisions (Leek & Johnston, 2009). Consequently, integrating these findings with the observed ERP results suggests that the MR process constitutes a high-level cognitive operation predominantly localized to the fronto-central region, where significant sex differences were identified. The larger amplitude observed in females suggests a greater mobilization of cognitive resources during the MR process, resulting in prolonged response times for task completion.

Furthermore, prior research has indicated that females exhibit a greater propensity for employing partial and local strategies to complete mental rotation (MR) tasks, whereas males are more inclined towards holistic strategies. Some authors have proposed that these distinctions may reflect a more fundamental divergence, with males adopting a parallel “Gestalt” perceptual strategy and females utilizing a “sequential” reasoning strategy (Hugdahl et al., 2006). In the current investigation, we deliberately segmented the target object into two components presented across consecutive slides. Participants were instructed to mentally rotate and integrate these components via MR, subsequently compare the resultant mental image with objects stored in memory, and finally provide their responses. This task design was implemented to mitigate potential strategy adoption differences attributable to procedural variations. Nevertheless, significant gender differences persisted, suggesting that gender disparities in MR performance remain stable and exhibit a relatively weak association with strategic approach. The observed ERP components reflect differential manifestations of underlying neural activity. Finally, in conjunction with the prolonged response times evident in behavioral performance, these findings indicate that females exhibit greater engagement of prefrontal cognitive control networks during task execution, incurring a temporal cost, yet without significant differences in accuracy.

Analysis of functional connectivity (FC) strength revealed significant gender differences in beta- and gamma-band oscillations, with males demonstrating greater connection strength in both frequency bands. This finding corroborates previous research implicating frontal and parietal regions in visuospatial processing, highlighting the importance of their connectivity for supporting visuospatial operations (Jouzizadeh, 2024). The study identified significantly stronger beta- and gamma-band FC within the male cohort. Prior research has established that spontaneous mental imagery is modulated by beta-band activity (Kaufman et al., 1990). During MR involving visual imagery, beta-band oscillatory activity within frontal and parietal regions—specifically the inferior parietal lobule and intraparietal sulcus—is associated with the perception of object motion (Borst et al., 2012). Synthesizing these observations, the present results suggest that the superior MR performance observed in males may arise, in part, from enhanced connectivity within core visuospatial regions, which play a crucial role in facilitating the underlying cognitive processes.

Stronger connectivity within the fronto-parietal region in males was also identified in the gamma-band. Gamma-band activity plays a critical role in brain function, particularly in visual cognition (Hoogenboom et al., 2006; Lachaux et al., 2005), and it is considered to reflect the integration of information distributed across distant cortical regions (Bressler, 1990). Within the context of the mental rotation task, gamma-band activity assumes a crucial role (Engel & Singer, 2001; Wyart & Tallon-Baudry, 2008) and is modulated by individual differences in mental rotation performance (Iwaki et al., 2011; Jouzizadeh, 2024). Consequently, the significant differences observed within this frequency band provide further corroborating evidence that males demonstrate superior performance during the rotation task. It is plausible that enhanced connectivity and greater connection strength enable males to exhibit superior behavioral performance.

## 7. Conclusions

In summary, males demonstrate superior behavioral performance alongside reduced fronto-parietal event-related potential (ERP) activity and lower expenditure of mental resources and effort. However, they exhibit enhanced internal regulation reflected in connectivity patterns compared to females. This phenomenon may be attributable to the capacity of brain connectivity to assess the co-activation state between distinct aspects of brain activity. Task-based functional connectivity elucidates the regulatory mechanisms (or involvement) governing brain networks during task execution—specifically, how specific networks and structural connections are modulated. These networks and connection structures support the execution of specific cognitive tasks.

## Figures and Tables

**Table 1:** The significant differences in reaction time between groups.

Solution	Groups	M ± SD	Male vs. Female		
			t	*p*	95% CI
Successful	Male	3709.24 ± 958.58	−2.39	0.02 *	[−1105.96,−100.06]
	Female	4312.25 ± 1107.90			
Unsuccessful	Male	4540.74 ± 1213.38	−2.02	0.047 *	[−1186.25,−7.07]
	Female	5137.40 ± 1220.47			

Note. * <0.05; CI = Confidence interval of the difference.

## Data Availability

The datasets generated during the current study are available from the authors upon request.
